# Robotic intracorporeal bilateral ileal ureter replacement for the treatment of post-radiation complex ureteral stricture complicated by uretero-arterial fistula

**DOI:** 10.3389/fsurg.2026.1791415

**Published:** 2026-03-27

**Authors:** Tengfei Gu, Ting Chen, Yongtao Pan, Qinzhou Yu, Jie Li

**Affiliations:** Department of Urology, Lishui Municiple Central Hospital, The Fifth Afliated Hospital of Wenzhou Medical University, Lishui, China

**Keywords:** bilateral ureteral stricture, ileal ureter replacement, pelvic radiotherapy, robotic-assisted total intracorporeal surgery, uretero-arterial fistula

## Abstract

**Background:**

Radiotherapy is a crucial treatment modality for gynecological malignancies. However, managing its long-term complications, such as ureteral strictures and uretero-arterial fistulas, is challenging. These complications often lead to recurrent hematuria, infections, and renal function impairment. Traditional ureteral stent placement has limited efficacy, particularly for complex cases involving vascular complications, which frequently necessitate surgical intervention.

**Objective:**

To summarize the management of complex cases involving bilateral ureteral strictures combined with iliac artery aneurysms and uretero-arterial fistulas following radiotherapy for cervical cancer, and to explore the feasibility and clinical efficacy of robot-assisted laparoscopic bilateral ileal ureter replacement with bladder anastomosis.

**Materials and methods:**

The patient was a 73-year-old female who presented with bilateral ureteral strictures and recurrent hematuria 9 years after undergoing postoperative radiotherapy for cervical cancer. Previous treatments, including multiple ureteral stent placements and vascular interventions, had been ineffective. Imaging studies revealed strictures in the mid-to-distal segments of both ureters and an iliac artery aneurysm. Following a multidisciplinary team discussion, a robotic total intracorporeal bilateral ileal ureter replacement with bladder anastomosis was performed. A 25-cm segment of ileum was harvested and configured in an inverted “7” shape for the anastomosis, and an anti-reflux procedure was incorporated. Detailed surgical steps are available in the accompanying video.

**Results:**

The procedure was completed successfully, with a total operative time of 245 min and an estimated blood loss of approximately 80 mL, without conversion to open surgery. The patient recovered well postoperatively, with no complications such as anastomotic leakage or infection. The ureteral stents were removed 2 months after surgery. A follow-up examination at 3 months postoperatively revealed significant improvement: hydronephrosis had markedly decreased, serum creatinine levels had dropped from a preoperative value of 256 µmol/L to 93 µmol/L, urinary function was satisfactory, and the patient's quality of life had significantly improved.

**Conclusion:**

Robotic total intracorporeal bilateral ileal ureter replacement is a safe and effective method for treating complex post-radiation ureteral strictures complicated by vascular conditions. This approach significantly improves renal function and alleviates clinical symptoms, making it a viable option for complex cases that have failed conventional treatments.

## Introduction

Radiation therapy is a crucial treatment modality for gynecological malignancies, but the management of its long-term complications, such as ureteral strictures and uretero-arterial fistulas, remains challenging (1). Conventional treatments like ureteral stent placement often have limited efficacy and may even exacerbate uretero-arterial fistulas due to repeated interventions, potentially leading to life-threatening complications such as acute massive hemorrhage (2). This article reports a case of a patient with bilateral ureteral strictures, iliac artery aneurysm, and uretero-arterial fistula following radiotherapy for cervical cancer, who achieved favorable outcomes through robotic total intracorporeal bilateral ileal ureter replacement.

## Case presentation

A 73-year-old female patient was admitted on March 28, 2025, due to “bilateral ureteral stricture, status post percutaneous nephrostomy for 3 months.” The patient underwent radical hysterectomy for cervical cancer in 2016, followed by pelvic radiotherapy. In February 2022, bilateral hydronephrosis was identified on imaging, suggestive of bilateral ureteral strictures. Bilateral ureteral stents were placed and subsequently exchanged regularly. During the stent period, the patient experienced recurrent symptoms including urinary tract infections, lower back pain, and hematuria. Due to worsening hydronephrosis, the stents were later replaced with metal ureteral stents.

In August 2024, the patient developed refractory hematuria, which progressively worsened. A diagnosis of uretero-arterial fistula (UAF) was made. Multiple endovascular procedures with covered stent placements in the iliac vessels were performed, yet the hematuria remained poorly controlled. In December 2024, she experienced severe hematuria. Both ureteral stents were removed, and bilateral percutaneous nephrostomies were established concurrently with an additional vascular intervention.

**Figure 1:**
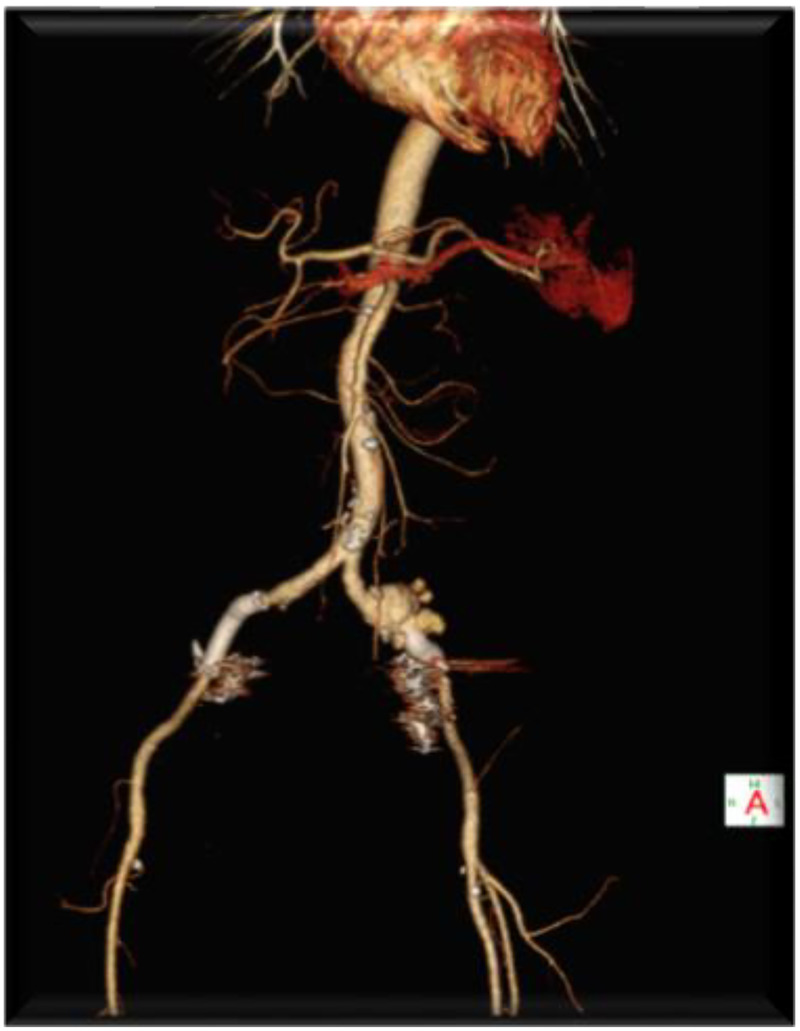
Bilateral iliac artery aneurysms.

In January 2025, the patient was transferred to our hospital due to severe hematuria resulting in bladder tamponade. On admission, her hemoglobin level was 45 g/L, accompanied by progressive renal impairment. Contrast-enhanced computed tomography (CT) revealed bilateral iliac artery aneurysms (Figure 1), with a maximum diameter of approximately 3.5 cm, confirming the diagnosis of UAF. Emergency management included endovascular intervention and bladder irrigation with clot evacuation.

**Figure 2:**
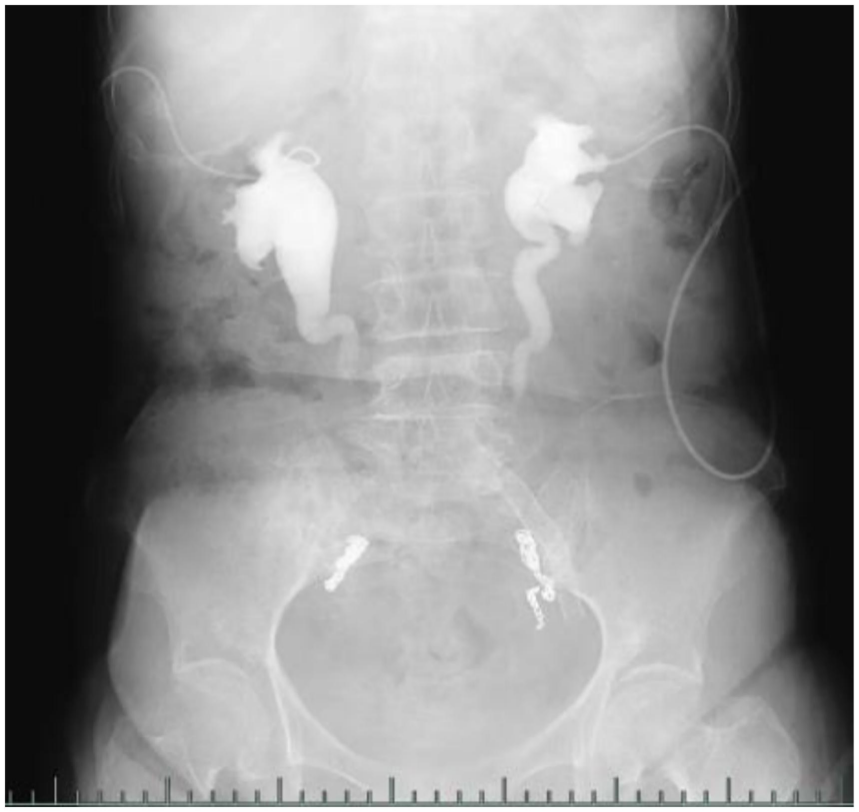
Urography indicates bilateral middle and lower ureteral stenosis, with a bladder capacity of approximately 300 mL.

**Figure 3:**
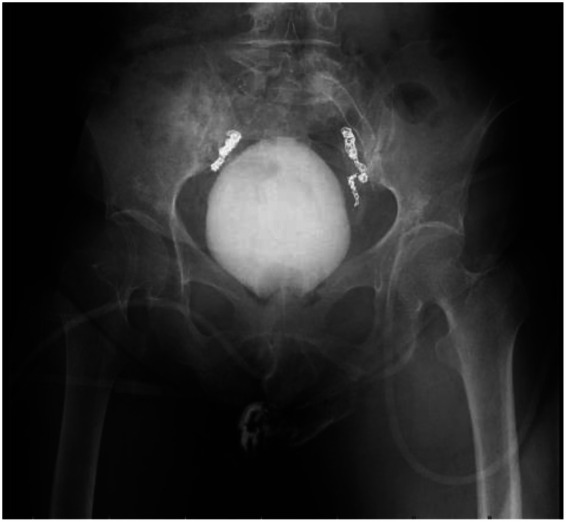
Urography indicates bilateral middle and lower ureteral stenosis, with a bladder capacity of approximately 300 mL.

In March 2025, following a multidisciplinary team (MDT) consultation, a definitive diagnosis of “post-radiotherapy bilateral ureteral strictures, bilateral iliac artery aneurysms, and uretero-arterial fistula” was established. As both interventional therapies and stent placements had failed, antegrade urography demonstrated strictures in the mid-to-distal segments of both ureters (approximately 8–10 cm in length) (Figure 2). Cystography indicated a bladder capacity of approximately 300 mL (Figure 3). It was decided to proceed with a robot-assisted total intracorporeal inverted “7”-shaped bilateral ileal ureter replacement with bladder anastomosis.

### Surgical procedure

**Figure 4:**
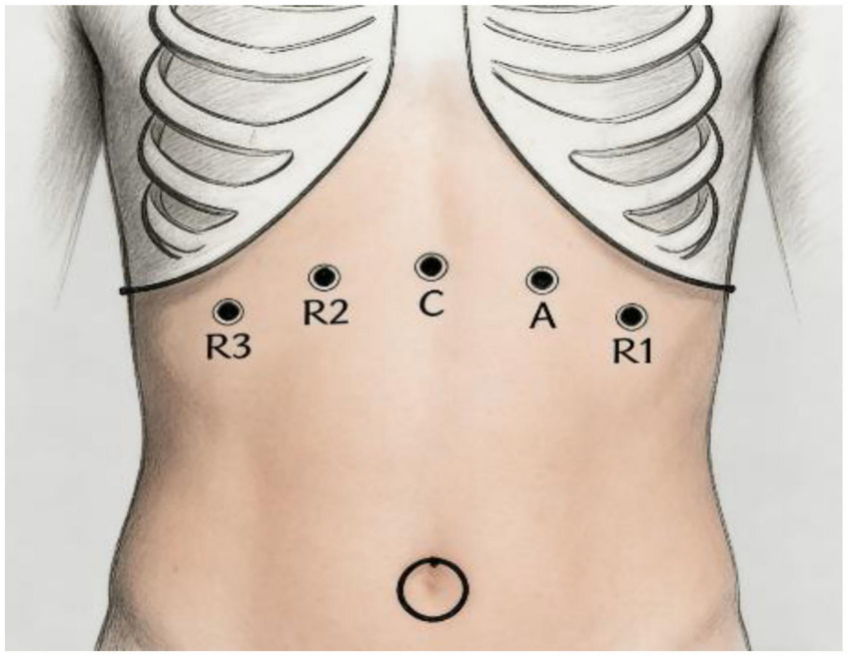
Port placement as illustrated.

The patient was placed under general anesthesia in the Trendelenburg position, with port placement as illustrated (Figure 4). Severe abdominal adhesions were encountered during the procedure and were meticulously lysed. Pelvic tissues were notably adherent, rendering the middle and lower segments of both ureters non-dissociable. An avascular window was created in the colonic mesentery to access the retroperitoneum, allowing for mobilization of the left mid-to-upper ureter. The left ureter was transected at a site with healthy vascular supply. The right ureter was managed using an identical approach to ensure optimal blood flow. A 3-cm longitudinal incision was made at the distal end of the left ureter, while the distal right ureter was spatulated for 2.5 cm.

Approximately 25 cm of ileum proximal to the ileocecal valve was isolated. A modified side-to-side ileal anastomosis was performed at the proximal and distal ends of this segment using a linear stapler-cutter along the anti-mesenteric border. The open end of the ileal segment was closed transversely with the stapler-cutter, and the staple lines were reinforced with 4-0 V-Loc sutures. The mesenteric defect was subsequently closed.

The distal end of the isolated ileal segment was intussuscepted upwards for approximately 2 cm and secured with full-thickness seromuscular sutures to fashion an anti-reflux nipple valve. Two F7 single-J ureteral stents were placed. One stent was externalized through the proximal end of the ileal segment according to the measured distance from the left renal pelvis, while the other was externalized through the side wall of the ileal segment based on the right-side measurement. The single-J catheters were advanced into the upper ureters and sutured to the intestinal wall for fixation.

**Figure 5:**
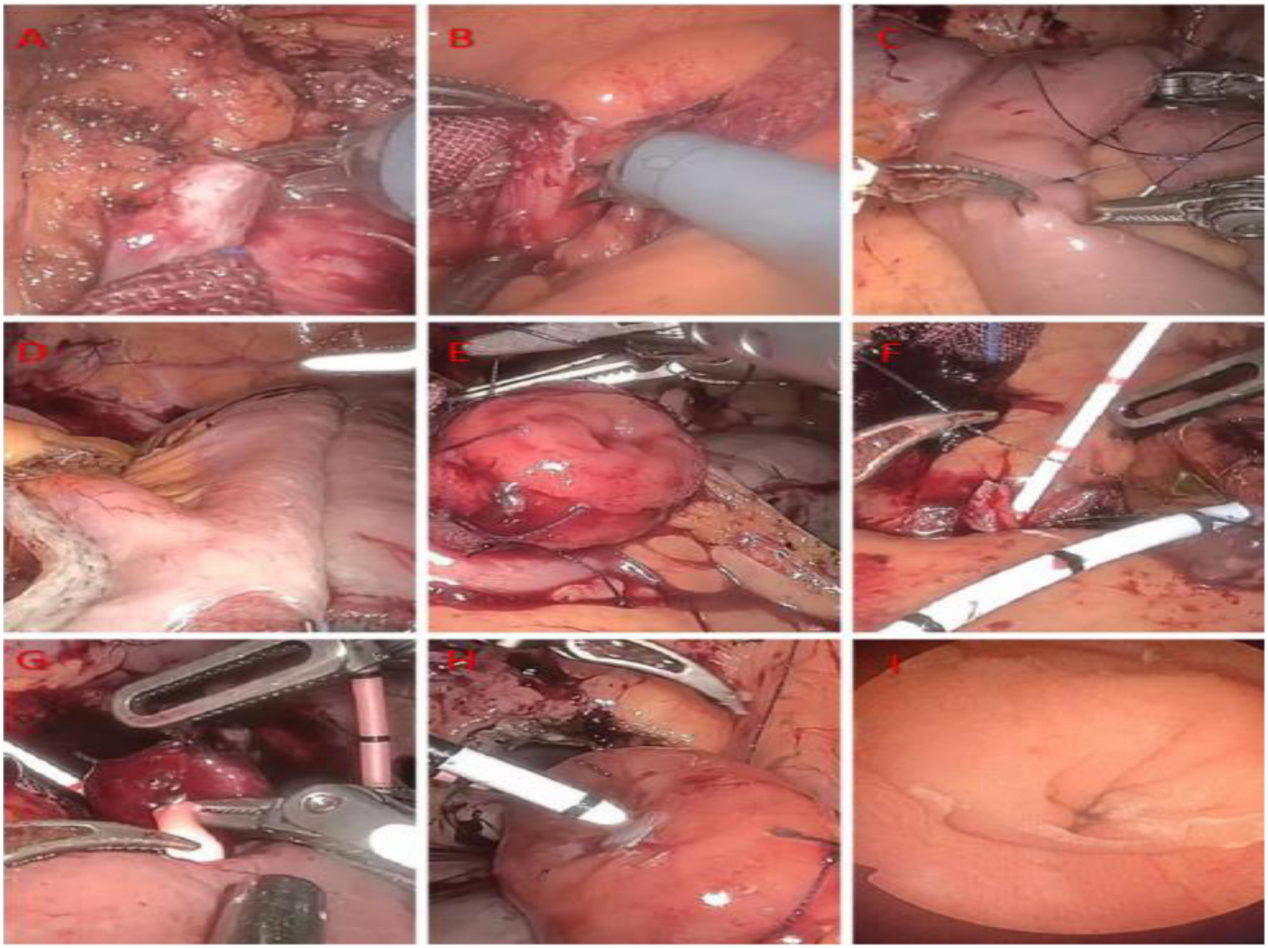
Surgical procedure for bilateral ureteral replacement with ileum and bladder anastomosis. **(A)**. Dissection of the right ureter; **(B)**. Dissection of the left ureter; **(C)**. Selection and marking of the desired intestinal segment; **(D)**. Restoration of intestinal continuity using a stapling device; **(E)**. Creation of an ileal nipple; **(F)**. End-to-end anastomosis of the left ureter to the ileal segment; **(G)**. End-to-side anastomosis of the right ureter to the ileal segment; **(H)**. Anastomosis of the ileal segment to the bladder; **(I)**. Cystoscopic view of the ileal nipple.

A continuous end-to-end anastomosis between the left ureter and the ileum was performed using 4-0 V-Loc sutures. The right ureter was anastomosed to the ileal segment in an end-to-side fashion. The bladder dome was incised, and the anti-reflux nipple was re-implanted into the bladder, with the anastomosis completed using 4-0 V-Loc sutures. A final inspection was conducted to confirm hemostasis and the absence of leakage at all anastomotic sites. A drainage tube was placed in the pelvic cavity (Figure 5).

### Surgical outcomes

**Figure 6:**
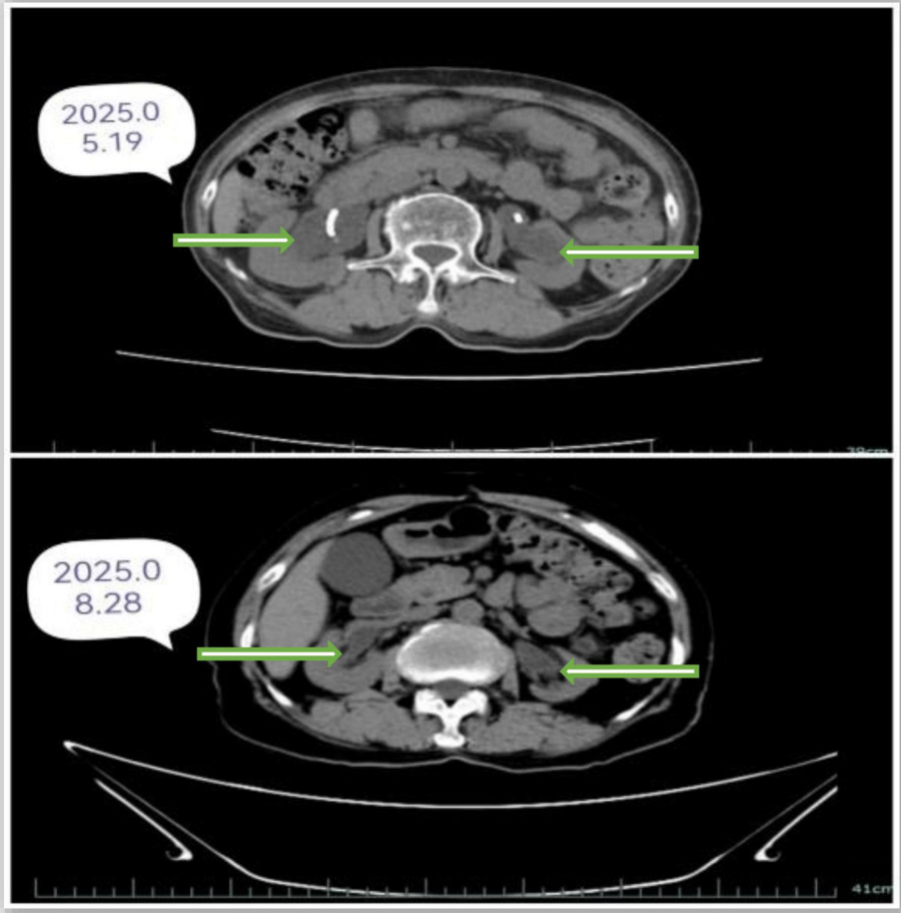
CT indicates improvement in hydronephrosis 3 months postoperatively.

**Figure 7:**
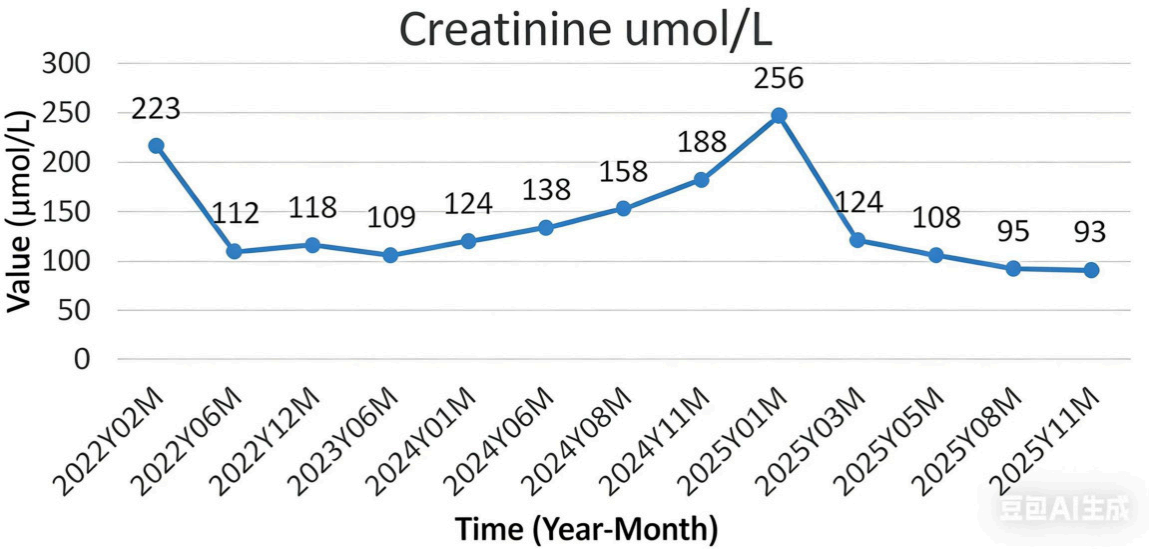
The patient's serum creatinine levels over the past 3 years have shown an improving trend postoperatively.

The procedure was successfully completed in 245 min with an estimated blood loss of 80 mL, without conversion to open surgery. The patient recovered well postoperatively, with no complications such as anastomotic leakage or infection. The drainage tube was removed at 1 week postoperatively, and the ureteral stents were removed at 2 months postoperatively. Follow-up examination at 3 months revealed significant alleviation of bilateral hydronephrosis (Figure 6). The serum creatinine level decreased from a preoperative peak of 256 μmol/L to 93 μmol/L (Figure 7). The patient demonstrated good voiding function and a significant improvement in quality of life.

## Discussion

This case involved a patient with bilateral complex ureteral strictures following pelvic radiotherapy for cervical cancer, complicated by an iliac artery aneurysm and ureteroarterial fistula (UAF). The condition was extremely complex. Traditional treatments, including repeated ureteral stenting and endovascular interventions, had failed. Ultimately, robotic-assisted complete intracorporeal bilateral ileal ureter replacement achieved a favorable outcome, offering a novel therapeutic strategy for such refractory cases.

In patients with post-radiation ureteral strictures, indwelling ureteral stents can predispose to UAF. The core mechanism lies in radiation injury, which leads to ischemia and fibrosis of the ureteral and adjacent vascular walls, rendering them rigid and fragile (3). On this pathological basis, the implanted stent creates persistent chronic mechanical friction and pressure within the stenotic lumen, potentially coupled with local inflammation, which collectively erodes the inelastic walls. Eventually, driven by arterial pressure, the rigid ureteral and vascular walls are penetrated, forming a direct communication and leading to life-threatening hemorrhage (4). The patient in this case also developed a UAF after multiple stent exchanges, culminating in massive, life-threatening bleeding.

Post-radiation pelvic tissues are characterized by severe fibrosis and adhesions, with compromised ureteral vascularity and intimate relationships with adjacent vessels, making direct stricture repair or ureteral anastomosis extremely challenging (5). Ileal ureter replacement utilizes an ileal segment with good blood supply and peristaltic function analogous to the ureter to bridge the long, diseased segment, effectively restoring urinary tract continuity (6). Compared to traditional open surgery, robotic-assisted complete intracorporeal operation offers advantages such as superior visualization, precise manipulation, minimal invasiveness, and reduced blood loss, making it particularly suitable for patients with severe pelvic adhesions and complex anatomy (7). The successful completion of the entire procedure intracorporeally without conversion to open surgery in this case with significant adhesions demonstrates the feasibility and safety of this technique.

Potential urine reflux following ileal ureter replacement, due to pressure transmission from peristalsis or elevated intravesical pressure, can lead to recurrent pyelonephritis and renal impairment. In this case, an anti-reflux nipple valve was deliberately constructed and implanted into the bladder, effectively reducing the risk of postoperative reflux and providing crucial protection for renal function. Additionally, the inverted “7”-shaped ileal anastomosis technique we employed, leveraging antegrade peristalsis, further mitigates the risk of urinary reflux (8).

Such patients often present with concomitant vascular complications, as seen here with the iliac artery aneurysm and UAF, rendering purely urological surgery extremely high-risk. Although preoperative endovascular intervention did not provide a definitive solution, it created a surgical window by controlling acute bleeding and stabilizing the patient. The entire diagnostic and therapeutic process was underpinned by multidisciplinary collaboration involving urology, vascular interventional radiology, imaging, oncology, and anesthesiology, which was fundamental to the successful execution of such a high-difficulty procedure.

The significant preoperative elevation in serum creatinine indicated severely compromised renal function. The normalization of serum creatinine and marked reduction of hydronephrosis at the 3-month follow-up confirmed the significant efficacy of this surgery in restoring upper urinary tract drainage and preserving renal function. This not only resolved the ureteral obstruction but also spared the patient from long-term dependence on nephrostomy or dialysis, substantially improving quality of life (9).

Despite the favorable outcome, it is crucial to acknowledge the specific limitations and potential complications of ileal ureter replacement in this patient population. Due to extensive pelvic fibrosis, compromised vascularity, and impaired healing capacity caused by radiation therapy, the risks of anastomotic leakage and stricture are higher compared to non-irradiated patients. Furthermore, the potential effects of radiation on the bowel itself may increase the risk of postoperative abdominal complications such as ileus or enteric fistula. Surgical success is highly dependent on meticulous dissection within the irradiated field, accurate assessment of blood supply, and excellent anastomotic technique. Therefore, stringent patient selection and meticulous perioperative planning are the cornerstones of a successful outcome. Future studies with larger case series and long-term follow-up are warranted to further elucidate the long-term efficacy and risks of this procedure in this complex, post-radiation cohort.

In conclusion, robotic-assisted complete intracorporeal bilateral ileal ureter replacement is an effective modality for managing complex post-radiation ureteral strictures complicated by vascular issues. It is particularly suitable for cases where traditional interventional and stent therapies have failed. Its advantages include precision, minimal invasiveness, and the ability to concurrently address long-segment strictures and the need for anti-reflux mechanisms. With multidisciplinary support, it can be performed safely and leads to significant improvements in renal function and patient quality of life.

## Data Availability

The original contributions presented in the study are included in the article/Supplementary Material, further inquiries can be directed to the corresponding author.
